# Environmental Surveillance through Machine Learning-Empowered Utilization of Optical Networks

**DOI:** 10.3390/s24103041

**Published:** 2024-05-10

**Authors:** Hasan Awad, Fehmida Usmani, Emanuele Virgillito, Rudi Bratovich, Roberto Proietti, Stefano Straullu, Francesco Aquilino, Rosanna Pastorelli, Vittorio Curri

**Affiliations:** 1 Department of Electronics and Telecommunications, Polytechnic University of Turin, 10129 Turin, Italy; fehmida.usmani@polito.it (F.U.); emanuele.virgillito@polito.it (E.V.); roberto.proietti@polito.it (R.P.); vittorio.curri@polito.it (V.C.); 2 School of Electrical Engineering and Computer Science (SEECS), National University of Sciences & Technology (NUST), Islamabad 45400, Pakistan; 3 SM-Optics, 20093 Cologno Monzese, Italy; rudi.bratovich@sm-optics.com (R.B.); rosanna.pastorelli@sm-optics.com (R.P.); 4 LINKS Foundation, 10129 Turin, Italy; stefano.straullu@linksfoundation.com (S.S.); francesco.aquilino@linksfoundation.com (F.A.)

**Keywords:** earthquakes, polarization, machine learning, early warnings, optical networks, sensing, waveplate model

## Abstract

We present the use of interconnected optical mesh networks for early earthquake detection and localization, exploiting the existing terrestrial fiber infrastructure. Employing a waveplate model, we integrate real ground displacement data from seven earthquakes with magnitudes ranging from four to six to simulate the strains within fiber cables and collect a large set of light polarization evolution data. These simulations help to enhance a machine learning model that is trained and validated to detect primary wave arrivals that precede earthquakes’ destructive surface waves. The validation results show that the model achieves over 95% accuracy. The machine learning model is then tested against an M4.3 earthquake, exploiting three interconnected mesh networks as a smart sensing grid. Each network is equipped with a sensing fiber placed to correspond with three distinct seismic stations. The objective is to confirm earthquake detection across the interconnected networks, localize the epicenter coordinates via a triangulation method and calculate the fiber-to-epicenter distance. This setup allows early warning generation for municipalities close to the epicenter location, progressing to those further away. The model testing shows a 98% accuracy in detecting primary waves and a one second detection time, affording nearby areas 21 s to take countermeasures, which extends to 57 s in more distant areas.

## 1. Introduction

Earthquakes represent one of the greatest natural disaster risks facing humanity. According to plate tectonics theory, the earth’s lithosphere is divided into plates by seismic zones that move relative to each other. The majority of earthquakes occur along these plates’ boundaries, with seismogenic faults being the geological origins of destructive earthquakes [1]. However, predicting earthquakes is a common scientific challenge for researchers globally. Much of this difficulty stems from the lack of reliable precursory indicators that meet the sufficient and necessary conditions for their occurrence, which is often considered the primary cause of failure in earthquake prediction efforts in earth science research. Monitoring these seismic events is an essential part in trying to predict them and employs a range of different methods. For instance, absolute measurements of geostress are used to assess the stress characteristics of significant faults [2], as seen in the San Andreas Fault Observatory at Depth (SAFOD) project [3]. Li Siguang, a pioneer of earthquake prediction in China, pointed out that an earthquake is a process of accumulation of stress on seismogenic faults. Real-time monitoring of geostress using tools like stress gauges can be leveraged to track changes in fault lines, providing insights into the release of seismic energy [4]. Crustal strain monitoring through strain gauges and GPS technology has been developed for seismic research and prediction as well [5,6]. Additionally, infrared monitoring methods can be used, as the infra-sound signal in the far field is found to be strong within two to eleven days before an earthquake with a magnitude of M7.0 or higher and its spectral characteristics are apparently different from other natural events [7].

Unfortunately, in 1988, seismologists in United States deployed a dense network of monitoring stations focused mainly on “surface strain monitoring”, in addition to tracking geomagnetic, geoelectric, ground-water level, and hydro-chemistry data, to predict an M6 earthquake occurring in the Park field near the San Andreas fault. Yet, the anticipated earthquake did not occur until 2004, 16 years later than expected, and the monitoring equipment failed to pick up any anomalies or precursors [8]. Similarly, in 1995, an M7 earthquake struck in Hanshin, Japan, killing more than 6500 people, where the high-density GPS network in place did not capture warning signals. Consequently, the scientific community has become increasingly sceptical about earthquake prediction. In March 1997, Robert J. Geller published a paper titled “Earthquakes cannot be predicted” in *Science* magazine, which reflected the prevailing opinion on earthquake prediction [9]. Therefore, it is crucial to address this challenge differently by adopting novel methods for widely distributed early detection systems capable of rapidly identifying an event to activate different mitigation strategies and minimize humanitarian and economic impacts. According to the International Association of Seismology and Physics of the Earth’s Interior (IASPEI), one of the main potential earthquake precursors is changes in strain rates, which are the rates at which the Earth’s crust stretches or compresses [10]. This is because such changes are indicative of stress accumulation in the Earth’s crust, potentially pointing towards an upcoming seismic event. Consequently, as optical fiber cables are buried underground, they too experience stretching or compression in response to the strain rate changes caused by seismic waves. The mechanical and optical properties of an optical fiber, as well as the physical properties of the light wave propagating inside it, change due to applied mechanical stresses and external disturbances. This trend opens the possibility of using the optical networks as a wide distributed network of sensors for environmental sensing, such as for earthquake detection or anthropic activity monitoring [11,12]. Essentially, there are two types of seismic waves: body waves (primary (P) waves and secondary (S) waves) that propagate through the earth’s interior and surface waves that propagate along the earth’s surface. Surface waves carry the greatest amount of energy and are usually the primary cause of destruction [13]. Detecting P waves that precede earthquake’s destructive waves allows for the swift initiation of emergency plans. Therefore, we have recently witnessed a rise in distributed fiber optic sensors that offer the possibility of measuring a slow-varying environmental variable at any location along the fiber length with a given sharp spatial resolution. This approach has been developed in the last decade to monitor dynamic strain variations induced by external perturbations using optical fibers. Distributed optical fiber sensors utilize the natural scattering processes arising in optical fibers, including Brillouin, Raman, and Rayleigh scattering. Rayleigh scattering combined with Optical Time-Domain Reflectometry (OTDR) or Optical Frequency-Domain Reflectometry (OFDR) has allowed the development of Distributed Acoustic Sensing (DAS) [14,15]. DAS employs an optoelectronic interrogator, which sends short light pulses into the fiber cable and then measures the optical perturbations in the light that scatters back, thereby deriving strain-rate signals proportional to the amount of physical stress impacting the fiber. These systems require dedicated “dark” fibers (i.e., optical fibers used solely for sensing without any communication channels) to operate [16,17,18], thus limiting the overall data-carrying capacity in the network. Moreover, these sensing techniques are incompatible with the inline optical amplifiers that are commonly found along optical fibers’ paths, and this is because the optical isolators inside the amplifiers block the backscattered DAS signals. Although these amplifications could be removed along dark fibers, which would lead to rampant signal attenuation, it is worth mentioning as well that the usable range of this technology is less than 100 km and requires powerful computational, storage, and processing capabilities that are generally only available in high-cost systems [19,20]. Frequency metrology interferometric techniques were introduced to overcome DAS’s usable range limitations. These techniques can measure the femtosecond delays experienced by the light from an ultrastable low-phase Fabry–Pérot laser traveling through a fiber at a micrometer scale over several thousands of kilometers [21], but they are still interferometric techniques considered to use dedicated and expensive hardware. In this manuscript, we present a novel technique that employs light polarization sensing. Unlike DAS and interferometric systems, state-of-polarization (SOP) sensing based on machine learning (ML) analyzes the integrated polarization alterations in the modulated light traveling through traffic-carrying optical fibers [22]. Our approach aims to leverage interconnected terrestrial optical mesh networks as a whole smart sensing grid to produce early anomaly warnings by identifying the arrival of earthquakes’ P waves without adding expensive equipment to the network, ensuring long-range measurements and not requiring dedicated dark fibers, thanks to the centralized design of our smart sensing grid optical network approach, which we detail in this work.

Due to the applied mechanical stress, the local refractive index of the fiber core changes, giving rise to birefringence. Birefringence leads to different propagation speeds of the optical wave along the x and y axes of the fiber core [23], which results in light polarization changes. Hence, SOP variations are dependent on disturbances applied to the fiber and can advantageously be used for sensing purposes, particularly because optical fiber communication networks have become pervasive and are widely deployed around the globe. In this paper, we aim to exploit optical networks beyond their conventional use, integrating real ground displacement data from seven earthquakes that occurred in the Modena region in Italy with magnitude values ranging from four to six to train and validate an ML model. The purpose is to then test the model against an earthquake within the same range of magnitude, utilizing interconnected optical mesh networks in three distinct municipalities that will confirm the arrival of the earthquake’s P wave, particularly through three sensing optical fibers placed precisely where three seismic stations were originally positioned for data collection in these distinct areas. In Section 2, we detail the methodology behind SOP data collection leveraging a waveplate model. Section 3 introduces the ML model training and validation, followed by Section 4, which presents the seismic network architecture and ML model testing results. Furthermore, we aim to showcase in Section 5 the triangulation methodology employed by the network controller overseeing all connected networks for accurate epicenter localization and fiber-to-epicenter distance measurements. Section 6 concludes the study.

## 2. Waveplate Model

In an ideal optical fiber, which is typically circular in shape, the silica glass from which it is made is isotropic. In the weakly guiding approximation, such an optical fiber supports the propagation of two degenerate orthogonal polarization modes. In general, the theoretical polarization characteristic of an optical pulse is represented by these two distinct modes. However, in reality, optical fibers are often birefringent due to construction imperfections that disrupt the fiber’s cylindrical symmetry, thus affecting the polarization. This means that in a fiber section that is small enough, the perturbation or the internal birefringence stemming from construction imperfections can be assumed to be spatially uniform [24].

**Figure 1:**
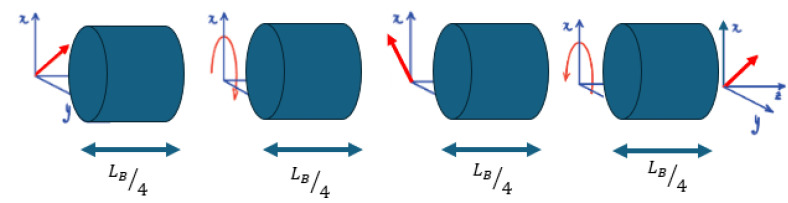
Schematic representation of fiber sections, each with uniform internal birefringence.

Seismic waves are another form of disturbances that cause external birefringence on the fiber and can also affect the polarization. To isolate and study the external disturbances on the light’s polarization within the fiber, it is crucial to understand the influence of internal birefringence. Here, adopting the waveplate model is essential to accurately define the effect of internal behavior by dividing the fiber into numerous small segments, referred to as ‘plates’, to ensure a uniform internal perturbed medium across each section [25]. Hence, the effect on light polarization is well defined and can be quantitatively described by 2π divided by the polarization beat length $L_{B}$, which is the amount of internal birefringence, defined as the propagation length over which the optical path length of the two polarization eigenmodes differs by exactly one wavelength, supplementary material of [24]. Consequently, any deviations from this established internal behavior can be attributed to external perturbations, as they would introduce unexpected changes in the state of polarization of light. Without considering any external effect, when linearly polarized light is injected at a 45-degree angle with respect to the linear polarization eigenmodes, the light acquires, after one quarter of $L_{B}$, a phase shift of $\frac{\pi }{2}$, transforming the linear input polarization into a circular one, and, after one half of $L_{B}$, it acquires a phase shift of π, as depicted in Figure 1. The waveplate model theory is described in Appendix A.

**Figure 2:**
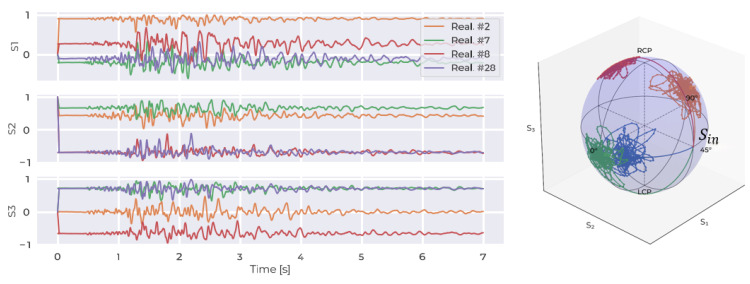
Four SOP evolutions for the same seismic event with different sets of plate angles.

However, by nature, these plates have random orientations, which cannot be controlled, adding complexity to the analysis of external effects. Basically, each plate is assigned with two random angles: ellipse of polarization or the major axis angle, and the eccentricity of the ellipse. For simplicity, in this paper, we only consider the major axis angle, which we present in Appendix A. In [25], the author presented the complete theory. Despite the random orientations of the plates causing varying polarization evolution, the data should contain invariant information linked to a specific earthquake. To overcome this complexity, a large evolution in polarization for a given seismic event is collected, where each SOP evolution corresponds to a different set of random plate angles, as in Figure 2, and a Monte Carlo simulation is carried out for these different random orientations. The goal of this is to train an ML model that can leverage this dataset to identify and understand the patterns in polarization changes that occur with the arrival of primary earthquake waves in order to detect the arrival of surface waves early.

This is where the ML model becomes valuable. Instead of analyzing the changes in the three Stokes parameters presented for each SOP evolution (S1, S2, S3), and to reduce computational time, we propose to calculate the state-of-polarization angular speed (SOPAS) [26] for each SOP evolution from their Stokes representations, which we detail in Appendix B. Thus, we analyze one variable instead of three. Moreover, one of the main functions of the Python-based waveplate model we have developed is converting earthquake ground displacement values into nanostrain values coupled to the fiber according to the conventional iDAS conversion presented in [27], where each 116 nm of ground displacement corresponds to a nanostrain fiber deformation of 11.6.

## 3. Machine Learning Model Training and Validation

Through the Italian National Institute of Geophysics and Volcanology (INGV) [28], we extracted real ground displacement data from seven local earthquakes recorded in the Modena region. The magnitudes of the chosen earthquakes were M4, M4.3, M4.5, M4.7, M5.1, M5.3 and M5.8. The objective is to integrate strain values caused by these displacements into fiber cables, each of 10 km, simulated with the aforementioned waveplate model. Light polarized at 45 degrees was injected into the model to conduct 50 simulations for each earthquake, where each simulation was assigned to random plate orientations. The evolution of the SOP was captured for each setting and subsequently converted into SOPAS values, resulting in a total of 350 simulation files used for training and validating the ML algorithm across all earthquakes. A Temporal Fusion Attention Network (TFAN) based on a neural network architecture was utilized for ML modeling, in which we combined a Temporal Convolution Network (TCN) [29], Long Short-Term Memory (LSTM) [30], and an attention mechanism. The term “temporal” in the model’s name indicates the focus on temporal data, capturing patterns and dependencies over time. “Fusion” represents features from both TCM and LSTM layers. As for “Attention”, it highlights the utilization of attention mechanisms to dynamically weigh the importance of different steps.

**Figure 3:**
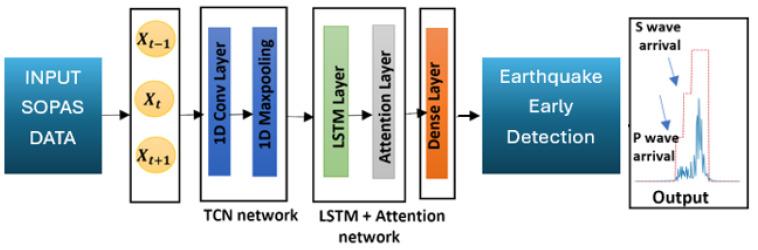
ML model architecture.

As shown in Figure 3, the model architecture is structured to process time-series data, followed by a TCN layer designed to capture temporal patterns. An LSTM layer is incorporated for long-term dependencies, with an attention mechanism to focus on significant time steps. The output layer facilitates multi-class classification with softmax activation. ML model training involves categorical cross-entropy loss, Adam optimization, and early stopping to mitigate over-fitting.

**Figure 4:**
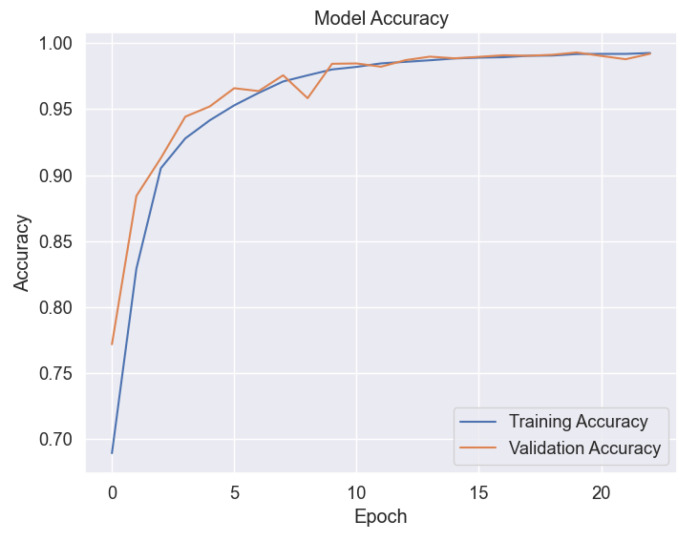
ML model training and validation accuracy.

Almost 60% of the SOPAS data were used for training, 20% were used for validation and 20% were used for testing. Figure 4 shows the model training and validation accuracy. The graph displays the accuracy over a sequence of epochs. The blue line represents the training accuracy, which increases rapidly, indicating effective initial training. Meanwhile, the orange line signifies the validation accuracy, assessing the model’s performance on new unseen data. The close alignment of these curves indicates that the model has been well generalized with minimal risk of over-fitting. As epochs progress, both curves reach noticeable accuracy rates, implying that further training is unlikely to yield significant improvements. The model shows a promising predictive precision, with the training and validation accuracy exceeding 95%.

## 4. Smart Sensing Grid Approach: Seismic Network Implementation

To manage the challenges of swiftly evolving traffic patterns, optical networks are evolving towards dynamically reconfigurable, autonomous systems. These systems are managed by a centralized Optical Network Controller (ONC), which interacts with Network Elements (NEs) by means of Application Programming Interfaces (APIs). The ONC leverages various metrics tracked by each NE, constituting the streaming telemetry paradigm for network management purposes. This setup facilitates the provision of varied services to the higher network layers. We propose to expand the streaming telemetry paradigm to integrate early earthquake detection services into the existing network. The streaming telemetry paradigm entails continuous data transmission from NEs to the ONC to assist network management and control. Devices like reconfigurable add/drop multiplexers (ROADMs) and amplifiers include crucial information like power levels and variations in temperature, whereas devices like coherent transceivers (TRXs) capture alterations in the phase and SOP of optical signals. External stress affects the phase and SOP of the transmitted signal; therefore, SOP changes carry environmental data that can be leveraged for sensing applications [31,32]. Furthermore, a post-processing agent within the NEs only filters the crucial information to the ONC and analyzes the data by leveraging machine learning algorithms. Coherent transceivers are inaccessible due to vendor lock, yet intensity-modulated direct-detected (IM-DD) TRXs are still popular in metro and access segments with lower data rates or function as slower Optical Supervisory Channels (OSCs) that terminate at every amplification site [33]. Thanks to the polarized nature of OSCs, the identification of OSC SOP alterations induced by external stress is facilitated. This can be achieved by extracting a minor portion of power to supply a polarimeter or a simple polarization beam splitter (PBS).

### 4.1. Case Scenario

**Figure 5:**
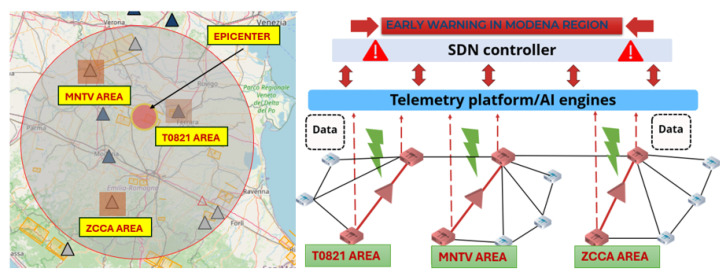
M4.3 earthquake time: 23 May 2012 21:41:18 (UTC). Region: Modena and corresponding interconnected sensing grid in the Modena region.

For testing the model, we used the M4.3 earthquake that occurred in the region of Modena on 23 May 2012. The objective of this is to leverage three interconnected terrestrial optical mesh networks in the region as a smart sensing grid driven by the aforementioned trained ML model, where we extracted the real ground motion data recorded by three seismic stations (T0821 at 23.14 km from the epicenter, MNTV at 47.88 km from the epicenter and MNTV at 61.45 km far from the epicenter), as shown in Figure 5.

Displacement values were then converted into strain values coupled along three sensing fibers positioned to correspond to the seismic stations’ geographical coordinates in the three distinct areas, where each fiber was divided into 2500 waveplates with a 4 m spatial resolution. NEs in each network will continuously send information to the ONC overseeing all mesh networks. We aim to confirm the event from three sensing areas, localize the epicenter and determine the epicenter to station/fiber substitute distance by applying a triangulation method that we detail in next section to generate early warnings accordingly. The ONC will confirm the event and issue early warnings after the third confirmation.

**Figure 6:**
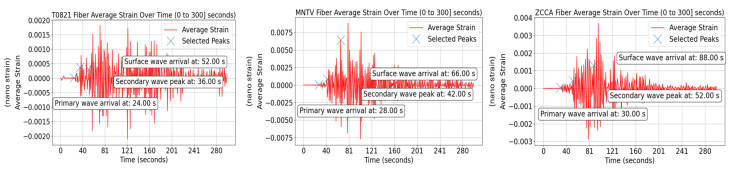
Strain evolution over the T0821 fiber (**left**), MNTV fiber (**middle**), and ZCCA fiber (**right**).

According to the Central Italian Apennines (CIA) velocity model [34], the time window between the primary wave and the arrival of surface wave increases with the increase in the distance from the epicenter, as does the primary wave arrival time. The earthquake struck at 21:41:18 UTC, and the P wave arrived at T0821 after 24 s, MNTV after 28 s and ZCCA after 30 s, as shown in Figure 6. Consequently, the P wave arrival time is 21:41:42 UTC at T0821, 21:41:46 UTC at MNTV and 21:41:48 UTC at ZCCA. The time window between the arrival of the P and surface waves at T0821 is 28 s (52 − 24), 38 s at MNTV (66 − 28) and 58 s at ZCCA (88 − 30). Thus, the arrival time of the surface wave is 21:42:10 UTC at T0821, 21:42:24 UTC at MNTV and 21:42:46 UTC at ZCCA.

We introduce detailed numbers to show that the time available for early warning in each area is as follows:

(We denote the ML P-wave detection time as MLDT and the time difference as TD)

In the T0821 area (seconds):

(1) $$\mathrm{Time}\;\left(\mathrm{seconds}\right)=21:42:10-\left(21:41:42+T0821_{\mathrm{MLDT}}+\left(P-\mathrm{wave}\;\mathrm{TD}\;\mathrm{with}\;ZCCA-T0821_{\mathrm{MLDT}}\right)+ZCCA_{\mathrm{MLDT}}\right)$$

In the MNTV area (seconds):

(2) $$\mathrm{Time}\;\left(\mathrm{seconds}\right)=21:42:24-\left(21:41:46+MNTV_{\mathrm{MLDT}}+\left(P-\mathrm{wave}\;\mathrm{TD}\;\mathrm{with}\;ZCCA-MNTV_{\mathrm{MLDT}}\right)+ZCCA_{\mathrm{MLDT}}\right)$$

In the ZCCA area (seconds):

(3) $$\mathrm{Time}\;\left(\mathrm{seconds}\right)=21:42:46-\left(21:41:48+ZCCA_{\mathrm{MLDT}}+\mathrm{Zero}\;\mathrm{knowing}\;\mathrm{that}\;ZCCA\;\mathrm{is}\;\mathrm{the}\;\mathrm{reference}\;\mathrm{station}\right)$$

It is good to note that the time difference of primary wave arrivals between T0821 and ZCCA is 6 s (30 − 24), and 2 s (30 − 28) between MNTV and ZCCA.

### 4.2. ML Model Testing Results

**Figure 7:**
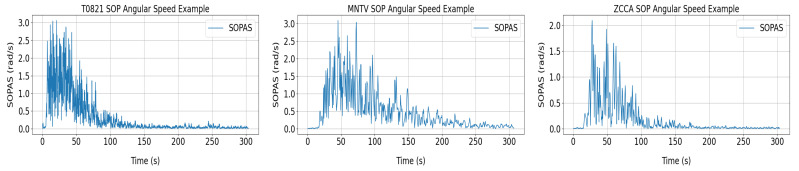
SOPAS evolution over the T0821 fiber (**left**), MNTV fiber (**middle**), and ZCCA fiber (**right**).

An example of one simulation for each sensing fiber substituting each seismic station is shown in Figure 7. SOPAS data on all fibers were utilized to test the trained ML model.

**Figure 8:**
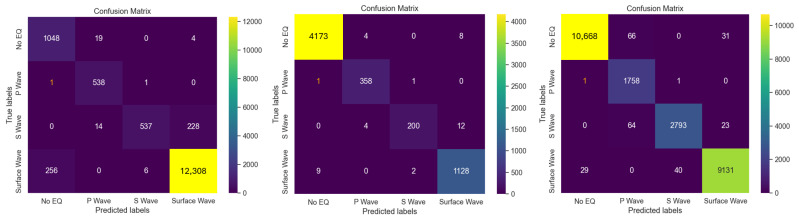
Confusion matrices over the T0821 fiber (**left**), MNTV fiber (**middle**), and ZCCA fiber (**right**).

We detail in this section the ML findings. We present in Figure 8 the confusion matrices for each fiber substitute, which show a table to visualize the performance of the classification model or seismic event classification system.

Each matrix is a measure of accuracy for predicting four categories: No EQ (no earthquake), P wave (primary wave), S wave (secondary wave) and surface wave. For the T0821 fiber substitute, presented on the left of Figure 8, it is shown that the system accurately detects ‘No EQ’ most of the time, with 1048 correct detections and 19 wrongly detected as P waves, 0 as S waves and 4 as surface waves. For P waves, it correctly identified 538 instances, missing only one as ‘No EQ’ and one as an S wave. Using this analysis with all matrices, there were 538 correct P wave detections out of 540 events for T0821 fiber substitute, 358 correct detections out of 360 for the MNTV fiber substitute (middle of Figure 8) and 1758 correct detections out 1760 for the ZCCA fiber substitute (right Figure 8). Consequently, the system modeling shows low false positive rates and a high level of efficiency with a 98% accuracy rate in detecting P waves, an essential component in early earthquake detection and seismic analysis.

**Figure 9:**
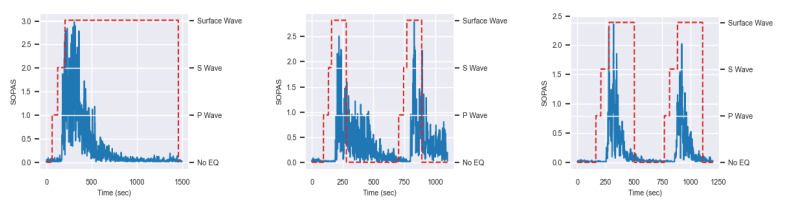
ML detection time of P waves using SOPAS data across three seismic stations/sensing fibers: T0821 (**left**), MNTV (**middle**) and ZCCA (**right**).

As mentioned earlier, the ML model utilizes SOPAS data as metrics for detecting and visualizing the presence of a seismic event. Employing one SOPAS example for the T0821 fiber substitute, two for MNTV and two for ZCCA, we show in Figure 9 the results of ML fitting for SOPAS data that demonstrate one-second of ML detection time over all fibers upon direct P wave arrivals, which means that if the P wave starts at t = 0, the detection will occur at t = 1. This implies that $T0821_{\mathrm{MLDT}}={\mathrm{MNTV}}_{\mathrm{MLDT}}={\mathrm{ZCCA}}_{\mathrm{MLDT}}=1$. As a result, Equations (1), (2) and (3) will become for T0821, MNTV, and ZCCA, respectively:

(4) $$\mathrm{Time}\;\left(\mathrm{seconds}\right)=21:42:10-\left(21:41:42+7\right)$$

(5) $$\mathrm{Time}\;\left(\mathrm{seconds}\right)=21:42:24-\left(21:41:46+3\right)$$

(6) $$\mathrm{Time}\;\left(\mathrm{seconds}\right)=21:42:46-\left(21:41:48+1\right)$$

Consequently, the time available for the T0821 area to take countermeasures is 21 s, and it is 35 s for the MNTV area and 57 s for the ZCCA area.

## 5. Triangulation Method for Localization Purposes

This method is employed by the ONC to pinpoint the earthquake’s epicenter and determine the station/fiber distance from the epicenter to generate early warnings for the nearest area to the epicenter and progress to those further away. The simulator uses measurements of seismic wave arrival times at different stations and their geographical coordinates to estimate the most probable epicenter location by minimizing the differences between expected and observed arrival times. The simulator defines the speed at which the seismic waves propagate through the Earth’s crust, and the coordinates and the exact time at which the wave was detected at each station are specified. The simulator then transforms the wave arrival at each station into seconds relative to the first recorded arrival, and a residual function is employed after to calculate the discrepancies between measured and theoretical expected arrival times. This is achieved by measuring the distance of each station from a hypothetical epicenter, converting these distances to expected times based on the seismic wave velocity, and then summing the squared differences to create an objective function for optimization. The simulator assumes an initial epicenter positioned at the centroid of the triangle formed by the three stations. A minimization function is then utilized to minimize the calculated residual sum.

Table 1 presents a comparative analysis between the actual seismic data recorded by INGV and theoretical detection from the triangulation simulator. The simulator shows almost identical measurements for both the epicenter latitude and longitude. Furthermore, the distances from seismic stations (T0821, MNTV and ZCCA) to the estimated epicenter location exhibit small discrepancies, and this is due to the fact that minor errors are picked up in the peaks from the graphs, and it could also be due to the displacement-to-strain conversion that affects the arrival times of P waves at each station. However, the triangulation method shows efficiency in its estimation. Consequently, the T0821 area is the first to be notified by the ONC about the upcoming seismic event, with a 21 s time lag for an emergency response before the surface wave strikes, followed by the MNTV area with a 35 s time lag and then the ZCCA area with a 57 s time lag.

## 6. Conclusions

In conclusion, this study shows the efficiency of using interconnected fiber optic mesh networks as a smart sensing and localization grid, leveraging machine learning for early earthquake detection. Real displacement data from seven earthquake events of varying magnitudes recorded by the INGV in the Modena region, Italy, were integrated into the model. This research showcases how the existing terrestrial fiber infrastructure can be utilized for accurate, real-time earthquake monitoring. The machine learning model developed and validated through this study not only improves the accuracy of earthquake detection and localization but also manages the distribution of early warnings. These capabilities represent a significant advancement in the geosciences area and earthquake response strategies, potentially reducing the impact on affected communities by providing time for critical responses. 

## Figures and Tables

**Table 1:** Comparison of epicenter locations and distances from seismic stations.

	Epicenter Location		Station to Epicenter Distance (km)		
	**Longitude**	**Latitude**	**MNTV**	**ZCCA**	**T0821**
INGV Recording	11.251	44.868	47.88	61.45	23.14
Triangulation Simulator	11.2846	44.8705	49.59	63.08	20.48

## Data Availability

The data presented in this study can be found at http://ismd.mi.ingv.it/ismd.php?tipo=lista (accessed on 20 April 2024).

## Abbreviations

SAFOD	San Andreas Fault Observatory at Depth
GPS	Global Positioning System
IASPEI	International Association of Seismology and Physics of the Earth’s Interior
P waves	Primary Waves
S waves	Secondary Waves
ML	Machine Learning
OTDR	Optical Time-Domain Reflectometer
OFDR	Optical Frequency-Domain Reflectometry
DAS	Distributed Acoustic Sensing
SOP	State of Polarization

## Abbreviations

SOPAS	State-of-Polarization Angular Speed
INGV	National Institute of Geophysics and Volcanology
CIA	Central Italian Apennines
ONC	Optical Network Controller
API	Application Programming Interface
NE	Network Element
ROADM	Reconfigurable Optical Add-Drop Multiplexer
TRX	Transceiver
OSC	Optical Supervisory Channel
IM-DD	Intensity Modulated Direct Detected
PBS	Polarization Beam Splitter
LSTM	Long Short-Term Memory
UTC	Coordinated Universal Time

## Appendix A. Waveplate Model Theory

A long telecommunication fiber is a good approximation of a concatenation of polarization waveplates with a random orientation and a random external birefringence. In a frequency interval in which the first-order approximation for the state of polarization is valid, any fiber section or waveplate is characterized by a Jones matrix.

(A1) $$M\left(\omega \right)=e^{j\beta (\omega )U(\omega )}=e^{j\beta (\omega )}\left(\begin{array}{cc} u_{1} & u_{2}\\ -u_{2}^{*} & u_{1}^{*} \end{array}\right)=e^{j\beta (\omega )}R_{\mathrm{out}}^{-1}M_{d}R_{\mathrm{in}}$$

where β is a quantity not essential for the calculation of the SOPs.

$$M_{d}=\mathrm{DIAG}\left(e^{j\omega ∆\tau /2},e^{-j\omega ∆\tau /2}\right)$$

where ω represents the difference between the generic frequency of the optical signal and the central frequency $\omega_{0}$, and ∆τ is the differential group delay (DGD) of the fiber and can be described as:

(A2) $$∆\tau =\frac{2\pi }{L_{b}}\left(1+\frac{∆L_{i}}{dz}\right)dz$$

where $\frac{2\pi }{L_{b}}$ is the internal birefringence presented earlier and $∆L_{i}$ is the external birefringence corresponding to the nanostrain value induced by an earthquake.

$M_{d}$ represents a rotation around a fixed axis through an angle equal to ω∆τ. $R_{\mathrm{in}}$ and $R_{\mathrm{out}}$ are matrices depending on the state of polarization and are described as:

(A3) $$R_{\mathrm{in}}=\left(\begin{array}{cc} \cos\theta & -\sin\theta \\ \sin\theta & \cos\theta \end{array}\right)$$

(A4) $$R_{\mathrm{out}}=\left(\begin{array}{cc} \cos\theta & -\sin\theta \\ \sin\theta & \cos\theta \end{array}\right)$$

The angle θ is the major axis angle that we consider in our model and the eccentricity of the ellipse is neglected. In [25], the full matrix representation was mentioned.

The matrix U(ω) of the cascade of two waveplates, described by $U_{1}$ and $U_{2}$, is

(A5) $$U=\left(U_{2},U_{1}\right)=\left(R_{\mathrm{out}2}^{-1}\left(M_{d2}\right)\left(R_{\mathrm{in}2}\right)\right)\left(R_{\mathrm{out}1}^{-1}\left(M_{d1}\right)\left(R_{\mathrm{in}1}\right)\right).$$

The more the fiber is segmented into waveplates the better, as this ensures small sections and a uniform internal birefringence in each section. Consequently, the polarization at the output of the fiber is calculated as

(A6) $$S_{\mathrm{out}}=U\times S_{\mathrm{in}}$$

## Appendix B. State-of-Polarization Angular Speed (SOPAS) Theorem

The state of polarization is the increase in the Stokes parameters samples taken at discrete time instants, represented by the vector *k* with components (S1[k],S2[k],S3[k]). The discrete state-of-polarization angular speed (SOPAS), denoted by ω[k], and the sampling period $T_{s}$ are given by the following relationship, where $\left(S_{k},S_{k-1}\right)$ is the dot product between the Stokes vectors at time *k* and time k−1. This computation is analogous to the discrete-time derivative of an angle, and the SOPAS is denoted by ω[k], where ω[k] is:

(A7) $$\omega \left[k\right]=\arccos\left(\frac{\left(S_{k}\cdot S_{k-1}\right)}{∥S_{k}∥∥S_{k-1}∥}\right)\cdot \frac{1}{T_{s}}$$
